# The mechanism of peer support on the mental toughness of adolescent swimmers: the mediating role of self-efficacy

**DOI:** 10.3389/fpsyg.2025.1663002

**Published:** 2025-10-27

**Authors:** Bingzhou Chen, Haixia Li, Ruiyun Zhang

**Affiliations:** 1 Graduate School of Education, Shandong Sport University, Jinan, China; 2 School of Sport Management, Shandong Sport University, Jinan, China; 3 College of Sports and Arts, Shandong Sport University, Jinan, China; 4 College of Physical Education and Health, East China Normal University, Shanghai, China

**Keywords:** peer support, mental toughness, self-efficacy, social cognitive theory, adolescent swimmers

## Abstract

**Objective:**

Current understanding of peer support’s role in swimmers’ mental toughness development remains limited. Guided by Bandura’s social cognitive theory, explores the relationship between peer support and the mental toughness of adolescent swimmers, along with self-efficacy’s mediating effect.

**Methods:**

This study used a quantitative cross-sectional design. A total of 161 adolescent swimmers, aged 10–18 years, participated in the research. Data on participants’ peer support, self-efficacy, and mental toughness were collected through questionnaire surveys. The data were analyzed using hierarchical linear regression, and mediation effects were tested with the bootstrapping method.

**Results:**

Studies have shown peer support, self-efficacy, and mental toughness showed significant intercorrelations. Mediation analysis confirmed that self-efficacy fully mediated the relationship between peer support and mental toughness: while peer support initially predicted mental toughness (*β* = 0.224, *p* < 0.01), its direct effect diminished (*β* = 0.107, *p* > 0.05) when self-efficacy (*β* = 0.418, *p* < 0.001) was included. The significant indirect effect via self-efficacy thereby highlights its role as the key mechanism through which peer support enhances mental toughness.

**Conclusion:**

Research has demonstrated that the presence of peer support is significantly positively correlated with mental toughness. Self-efficacy plays a mediating role between peer support and mental toughness.

## 1 Introduction

Mental toughness is a critical factor for success in competitive sports (Gucciardi, 2017). Competitive swimming places extremely high demands on athletes, such as undergoing extensive training, enduring tremendous pressure (Alvarez et al., 2021), and coping with the physical and mental fatigue associated with long-term aquatic conditioning (de Lima-Júnior et al., 2025). Therefore, in such high-pressure athletic contexts, the cultivation of mental toughness becomes paramount for sustained performance excellence (Nicholls et al., 2011; Zalewska et al., 2019). Bandura's (1986) social cognitive theory proposes a triadic reciprocal interaction among environmental influences, cognitive processes, and behavioral outcomes, wherein personal factors, behaviors, and environments bidirectionally shape one another (Bandura, 2001).

Mental toughness, characterized as a dynamic psychological resource enabling consistent high-level performance despite varying situational demands and adversities (Gucciardi, 2017; Gucciardi et al., 2015). This mental edge allows athletes to maintain focus, confidence, and goal pursuit while excelling under competitive pressures (Gucciardi et al., 2015), and enables adolescent swimmers to sustain self-confidence post-failure while actively recovering from performance troughs (Meggs and Chen, 2019). Beyond competitive advantages, mental toughness serves as a buffer for psychological well-being, enhancing swimmers’ motivation and training dedication while mitigating their exhaustion and burnout risks (Tian et al., 2022). Studies indicate that mental toughness varies by gender, age, and competitive level (Nicholls et al., 2009). Key influencing factors include team cohesion, social support, and resilience traits, with the satisfaction of basic psychological needs being significantly correlated with mental toughness development in young swimmers (Lou et al., 2014). The formation mechanisms of mental toughness are diversely interpreted. Hu and Gan (2008) process model frames mental toughness through two dimensions: personal strength including internal factors like goal focus and emotional control, and supportive force encompassing external factors like peer support and coach guidance.

Notably, self-efficacy refers to an individual’s belief in their capability to take specific actions to achieve set goals (Bandura, 1977, 1997), which aligns with the dimension of internal factors proposed by Hu and Gan (2008). Self-efficacy can enhance the motivation and performance of adolescent swimmers in their training (Wang et al., 2025). Studies also show that self-efficacy and mental toughness are strongly correlated (Ali et al., 2024), and sports contexts further strengthen this relationship (Rogowska et al., 2022).

Bandura’s (1986) social cognitive theory conceptualizes self-efficacy as its core. The present study positions self-efficacy as the mediator converting aquatic-specific peer interactions into mental toughness development. Social cognitive theory is grounded in human agency, the intentional capacity to regulate actions and modify environments to achieve goals (Bandura, 2001). This dual emphasis on contextual adaptability and proactive self-regulation explains validating the robustness of social cognitive theory in modeling adaptive behaviors across dynamic environments (Ramolale et al., 2021). Moreover, according to Bandura's (1986) social cognitive theory and previous research findings, self-efficacy is also influenced by peer support, which in turn affects mental toughness (Huang, 2023).

Peer support involves sharing emotions, experiences, and skills with age-similar peers sharing comparable life contexts (Moran et al., 2012). It encompasses beneficial impacts from peer interactions (Zou et al., 2023), motivates adolescent swimmers’ life and training choices (Finnerty et al., 2010), and creates supportive spaces for collaborative knowledge exchange and identity formation (Lee and Hung, 2024). Peer support exerts a significant influence on both the sport motivation and career burnout of adolescent swimmers (Alvarez et al., 2021). This phenomenon aligns with the support dimension outlined by Hu and Gan (2008).

While swimming is typically classified as an individual sport, swimmers do their training and even competitions as a team. These teenage swimmers spend a considerable amount of time at the training venue every day, resulting in them having frequent interactions with their teammates. This environment fosters social connections with teammates (Chan et al., 2012). In this situation, peer support becomes particularly important (Eccles, 1999). All athletes will receive support from their teammates, families, teachers and coaches, families and educators offer spoken social reinforcement, while peers engage in interactive social support (Pan et al., 2022). Examining peer support in adolescent swimmers helps clarify how aquatic training environments influence adaptive behavioral development, while contributing to social cognitive theory’s exploration of environmental inputs within its (Bandura, 2001). However, the question of how such increasingly frequent peer interactions during adolescence can shape mental toughness through water-based team training remains to be explored at present.

Adolescence is a crucial developmental stage where peer influence intensifies (Arnett, 1999; Berndt, 1979), making mental toughness a critical psychological resource for young athletes navigating future challenges (Chen et al., 2015). Basic psychological needs satisfaction further reinforces mental toughness (Mahoney et al., 2014), highlighting the interplay between supportive environments and mental toughness. In sports contexts peer derived social support enhances adaptive resilience and pressure management, synergizing with mental toughness to fortify competitive readiness (Laird et al., 2018). Research on adolescent swimmers has also examined knowledge, lifestyle, and behavioral factors influencing their development (AlKasasbeh et al., 2024; AlKasasbeh and Akroush, 2024). However, peer support effects vary significantly with team relational dynamics (Pacewicz et al., 2019). Despite adolescent swimmers extensive team exposure during training and competitions peer supports role remains underexplored. First prior research emphasizes individual traits (Chen and Cheesman, 2013; Gucciardi et al., 2015; Tian et al., 2022) or parental and coach influences (Nicholls et al., 2016; Vega-Díaz and González-García, 2024), while neglecting team-based peer interactions. Second existing work focuses on land-based projects with verbal encouragement (Khayati et al., 2024; Hammami et al., 2021), yet the exploration of aquatic training environments remains comparatively limited.

Bandura’s (1986) social cognitive theory provides an explanatory framework for competitive sports. Due to water based physical isolation swimmers rely on asynchronous and pre strategic peer support. Key mechanisms include pre race technical exchanges for example relay handoff optimization post training video analysis and post race debriefings. Through systematic integration of these peer interactions swimmers develop cognitive models for challenge management enhancing mental toughness via repeated peer engagement and experiential accumulation.

Kumalasari (2023) demonstrated that peer support in academic settings enhances students’ capacity to overcome academic difficulties and adapt to severe challenges. A similar effect may also exist in a sports environment.

For adolescent swimmers, peer support functions as an environmental input that elevates self-efficacy, a core cognitive process. This enhanced self-efficacy subsequently fosters mental toughness, the adaptive behavioral outcome. While the present study focuses on the directional pathway from environmental input to adaptive outcome, the triadic structure inherently aligns with SCT’s principle of dynamic interdependence (Bandura, 2001).

Based on Bandura’s (1986) social cognitive theory, self-efficacy mediates the peer support-mental toughness relationship through vicarious experiences and verbal persuasion. When adolescent swimmers observe teammates overcoming challenges or receive affirming peer evaluations, they internalize “I can do it” beliefs, enhancing self-efficacy. Yu et al. (2024) pointed out in their research, athletes who demonstrate a higher degree of mental toughness usually perform better under pressures. This heightened self-efficacy directly translates to mental toughness, enabling athletes to navigate competitive demands.

Critically, however, the absence of such mental toughness carries significant risks for athletic development. Insufficient mental toughness may not only contribute to a decline in competitive performance among adolescent swimmers, but also significantly impair their capacity to cope with adversity, adversely affect their decision-making under pressure, and diminish motivation, among other negative outcomes (Meggs and Chen, 2018; Moreira et al., 2021; Anthony et al., 2020). Given these challenges, the present study operationalized the core tenets of Bandura’s theory into a tailored framework specific to competitive swimming. The mediating role of self-efficacy in the relationship between peer support and mental toughness was delineated, thereby informing the development of evidence-based targeted training regimens and psychological interventions for coaches and sports psychologists to foster athletes’ adaptive capacities. In light of the aforementioned evidence, this paper proposes the research hypothesis:

H1: Peer support significantly predicts mental toughness in adolescent swimming athletes.

H2: Self-efficacy plays a mediating role between peer support and the mental toughness of adolescent swimmers.

## 2 Materials and methods

### 2.1 Research design

Based on the social cognitive theory, the present study employs a quantitative cross-sectional approach to investigate how peer support within swimming-specific environments enhances mental toughness among adolescent competitive swimmers through the mediating role of self-efficacy. The participants consist of youth athletes engaged in professional swim team training programs, typically involving 10–12 sessions per week, each lasting 2.5 h.

The study operationalizes its key variables as follows: peer support is conceptualized as a multifaceted environmental factor encompassing emotional, informational, and motivational exchanges within the swim team setting; self-efficacy represents the central cognitive mediator, reflecting athletes’ beliefs in their capabilities to perform and persevere under demanding training conditions; and mental toughness is regarded as the critical outcome variable, characterized by the ability to maintain focus, manage pressure, and recover effectively from setbacks in both training and competition.

**Figure 1:**
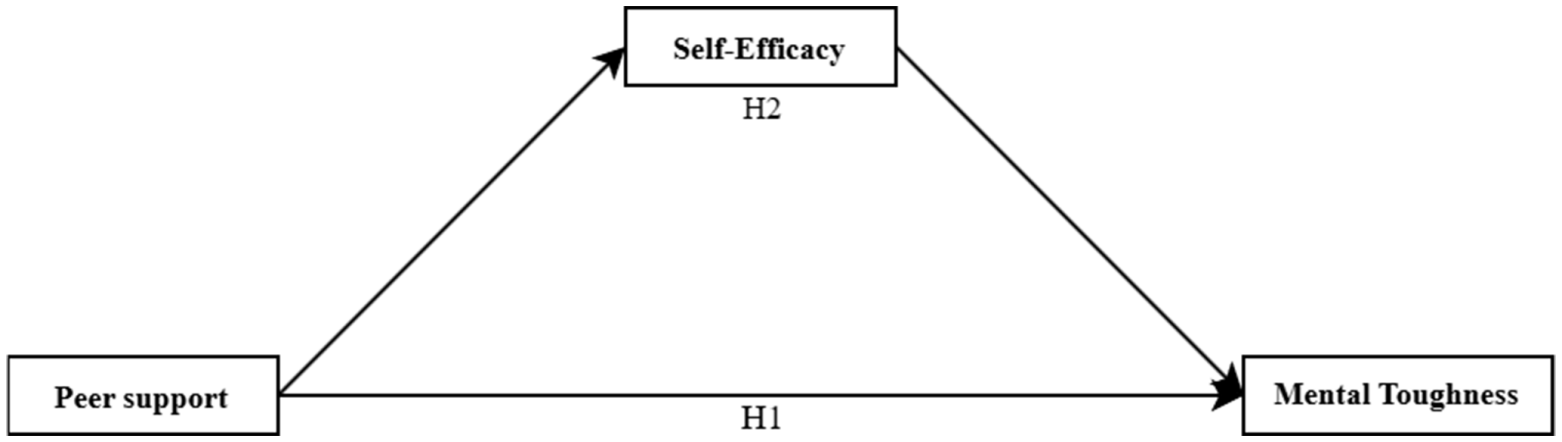
A hypothetical model of peer support affecting mental toughness.

The mediation model, which synthesizes the research, is presented in Figure 1.

### 2.2 Participants

A total of 170 athletes aged 10–18 years from 3 municipal swimming teams in Shandong Province participated in this study. After screening, 9 invalid questionnaires were excluded, comprising 5 due to incomplete responses, 3 failing to meet the minimum age requirement of 10 years, and 1 exceeding the maximum age limit of 18 years. A total of 161 valid samples were retained, yielding a retention rate of 94.7%. The sample comprised 70 males and 91 females, with a mean age of 14 ± 2.5 years, categorized into three age groups: 10–12 years (*n* = 56), 13–15 years (*n* = 50), and 16–18 years (*n* = 55). Training experience spanned four tiers: <1 year (*n* = 6), 1–3 years (*n* = 52), 4–6 years (*n* = 62), and >7 years (*n* = 41). Athlete grades included non-elite athlete (*n* = 27), second grade athlete (*n* = 74), first grade athlete (*n* = 52), and national master of sports (*n* = 8).

### 2.3 Procedures

Prior to the survey, a contextualized scenario was designed to simulate an ecologically valid swimming-specific environment, aligning with our social cognitive theory framework. This approach helped operationalize the key variables under study. Participants were asked to imagine having just finished a typical 2.5-h training session with peers, with a championship scheduled in 14 days. This scenario reflects the high-performance context of the sampled swimmers and effectively elicits the target constructs: peer support, mental toughness, and self-efficacy. Well-validated scales were used to measure these constructs, selected based on their alignment with our variable definitions and established reliability for athletic populations. To control for order effects, we administered the questionnaire using Questionnaire Star’s randomization feature, which varied item order across participants while keeping content consistent.

### 2.4 Data collection

#### 2.4.1 Peer support assessment

The peer support scale was adopted from the 10-item single-factor scale validated by Hsu et al. (2011) for adolescent populations, which originated from the Social Support for Exercise Scale (Sallis et al., 1987), to measure peer support for mental toughness. An example of one item is, “My friends gave me encouragement to stick with my swimming training” (see Table 1 for details). A 7-point Likert scale was used ranging from 1 (strongly disagree) to7(strongly agree). Reliability tests demonstrated robust internal consistency for the refined scale, with Cronbach’s *α* = 0.81, composite reliability (CR) = 0.88, and average variance extracted (AVE) = 0.70.

**Table 1:** Scale items, Cronbach’s alpha coefficients, and source references.

Variable	Item	Cronbach’s *α*	AVE	References
Peer support	My teammates gave me encouragement to stick with my swimming training	0.81	0.70	Hsu et al. (2011)
	My teammates gave me helpful reminders to engage in swimming training			
	My teammates discussed swimming techniques with me			
Mental toughness	I believe in my ability to achieve my goals	0.87	0.62	Gucciardi et al. (2015)
	I am able to regulate my focus when performing tasks			
	I consistently overcome adversity			
	I effectively execute my ability of what is required to achieve my goals			
	I am able to execute appropriate skills or knowledge when challenged			
Self-efficacy	If I do my best, I’ll always be able to finish even the most difficult training tasks.	0.88	0.67	Yu et al. (2024)
	For me, sticking to my ideals achieve the goals in terms of swimming performance is a sure thing			
	With my talents, I’ll be able to handle the unexpected in the competition.			
	If I put in the necessary effort, I am bound to be able to solve most of the puzzles in training.			

#### 2.4.2 Mental toughness assessment

The Mental Toughness Index developed by Gucciardi et al. (2015) was adopted, which asks athletes to assess the degree to which each item in this scale reflects their own thinking patterns, feelings, and behaviors in their sport. For example, “I believe in my ability to achieve my goals” (see Table 1 for details). Reliability tests demonstrated robust internal consistency for the refined scale, with Cronbach’s *α* = 0.87, composite reliability (CR) = 0.89, and average variance extracted (AVE) = 0.62.

#### 2.4.3 Self-efficacy assessment

The scale used in this study was derived from the work of Yu et al. (2024), who updated and validated the General Self-Efficacy Scale. The purpose of this scale is to measure the self-efficacy of individuals in various tasks, and it has been proven to be brief, easy to use, reliable and effective. This questionnaire has a unidimensional structure and is used to measure the overall and generalized self-efficacy of individuals. For example, “If I do my best, I’ll always be able to finish even the most difficult training tasks” (see Table 1 for details). Reliability tests demonstrated robust internal consistency for the refined scale, with Cronbach’s *α* = 0.88, composite reliability (CR) = 0.89, and average variance extracted (AVE) = 0.67.

#### 2.4.4 Data analysis

All statistical analyses were conducted using IBM SPSS Statistics 29.0 (Released 2022, Armonk, NY: IBM Corp).

To examine the predictive relationships among variables, hierarchical multiple linear regression analyses were performed using IBM SPSS Statistics 29.0. The analysis employed a three-step hierarchical approach: Model 1 included control variables (gender, age, training years, athlete grade); Model 2 added peer support to assess its direct effect on mental toughness; Model 3 further incorporated self-efficacy to evaluate its mediating role. All models used enter method for variable inclusion.

To test the mediating role of self-efficacy between peer support and mental toughness, Model 4 (simple mediation) analysis was conducted using the SPSS PROCESS macro with 5,000 bootstrap resamples (95% bias-corrected confidence intervals), controlling for gender, age, training years, and athlete grade.

## 3 Results

### 3.1 Measurement model assessment and variable characteristics

Characteristics Confirmatory factor analysis (CFA) was used to evaluate the measurement model. Descriptive, composite reliability, and correlation analyses were performed on all constructs. All constructs were measured on a 7-point Likert scale (1 = strongly disagree, 7 = strongly agree), with peer support showing a mean score of (*M* = 5.38, *SD* = 1.56), mental toughness (*M* = 5.34, *SD* = 1.46), and self-efficacy (*M* = 5.02, *SD* = 1.66) all demonstrating moderately high scores. Composite reliability (CR) values exceeded the threshold of 0.70 for all constructs: peer support (CR = 0.88), mental toughness (CR = 0.89), and self-efficacy (CR = 0.89) (Hair et al., 2010). Significant correlations (*p* < 0.01) were observed: peer support strongly correlated with mental toughness (*r* = 0.23) and self-efficacy (*r* = 0.28), while mental toughness and self-efficacy showed the strongest correlation (*r* = 0.46). These results provide preliminary support for H1. Complete statistical details are provided in Table 2.

**Table 2:** Descriptive statistics, composite reliability, and validity assessment.

Variable	M	SD	CR	PS	MT	SE
Peer support	5.38	1.56	0.88	**0.84**		
Mental toughness	5.34	1.46	0.89	0.23**	**0.79**	
Self-efficacy	5.02	1.66	0.89	0.28**	0.46**	**0.82**

PS, peer support; MT, mental toughness; SE, self-efficacy; CR, composite reliability.

*p < 0.05, **p < 0.01, ***p < 0. 001.Diagonal values in bold represent the square root of Average Variance Extracted (AVE).

### 3.2 Hierarchical regression analysis

A hierarchical regression analysis was performed to examine the predictors of mental toughness. In Model 1 (gender, age, training years, athlete grade), only age (*β* = 0.23, *t* = 2.07, *p* < 0.05) and training years (*β* = −0.23, *t* = −2.04, *p* < 0.05) significantly predicted mental toughness, with *R*^2^ = 0.04 (adjusted *R*^2^ = 0.02). Adding peer support in Model 2 increased *R*^2^ to 0.09 (Δ*R*^2^ = 0.05), peer support emerged as a significant predictor (*β* = 0.22, *t* = 2.91, *p* < 0.01), while age remained significant (*β* = 0.22, *t* = 2.02, *p* < 0.05). Further including self-efficacy in Model 3 increased *R*^2^ to 0.25 (Δ*R*^2^ = 0.16, *p* < 0.001), with self-efficacy as the strongest predictor (*β* = 0.42, *t* = 5.71, *p* < 0.001). Peer support’s effect became non-significant in Model 3 (*β* = 0.11, *t* = 1.46). These results indicate that self-efficacy fully mediates the effect of peer support on mental toughness. These results indicate that self-efficacy fully mediates the effect of peer support on mental toughness, supporting H1 and providing preliminary evidence for H2. Complete statistical details are provided in Table 3.

**Table 3:** Hierarchical regression analysis.

Variables	Model 1	Model 2	Model 3
Gender	−0.04 (−0.46)	−0.05 (−0.60)	−0.05 (−0.67)
Age	0.23* (2.07)	0.22* (2.02)	0.21* (2.06)
Years of training	−0.23* (−2.04)	−0.21 (−1.92)	−0.16 (−1.60)
Athlete level	−0.06 (−0.57)	−0.05 (−0.45)	−0.07 (−0.78)
Peer support		0.22** (2.91)	0.11 (1.46)
Self-efficacy			0.42*** (5.71)
*R* ^2^	0.04	0.09	0.25
Adjusted *R*^2^	0.02	0.06	0.22
*F*	1.64	3.07*	8.52***

*p < 0.05, **p < 0.01, ***p < 0.001, values in parentheses represent *t* statistics. The dependent variable is mental toughness.

### 3.3 Mediation analysis

Mediation analysis confirmed that the total effect of peer support on mental toughness was significant [*β* = 0.21, 95% CI (0.07, 0.35)]. The direct effect became non-significant after controlling for self-efficacy [*β* = 0.10, 95% CI (−0.04, 0.24)], while the indirect effect through self-efficacy remained significant [*β* = 0.11, 95% CI (0.04, 0.20)]. The absence of zero in the indirect effect’s CI and the non-significant direct effect confirm full mediation, thereby validating Hypothesis H2. Complete statistical details are provided in Table 4.

**Table 4:** Mediation analysis results.

Path	Effect type	SE	95%CI		
			Lower	*β*	Upper
Peer support → mental toughness	Total	0.07	0.07	0.21	0.35
Peer support → mental toughness	Direct	0.07	−0.04	0.10	0.24
Peer support → self-efficacy → mental toughness	Indirect	0.04	0.04	0.11	0.20

Indirect effect significance determined by CI exclusion of zero (Model 4). *β*: standardized regression coefficient. SE: Standard error. CI: Bias-corrected bootstrap confidence interval (5,000 resamples). Significance is inferred if the 95% CI excludes zero.

## 4 Discussion

Guided by Bandura’s (1986) Social Cognitive Theory, this study aims to investigate the mechanism through which peer support influences mental toughness among adolescent swimmers. The primary objective is to examine the mediating role of self-efficacy in this relationship, thereby enhancing adolescent swimmers’ mental toughness in high-pressure competitive environments. The findings and implications will be discussed in detail in the following section.

### 4.1 The relationship between peer support and mental toughness

The particular effectiveness of peer support in swimming may stem from the sport’s unique training structure, where adolescent swimmers train, compete, and spend prolonged time together, creating natural opportunities for observational learning and mutual encouragement. The repetitive and demanding nature of swim training creates frequent opportunities for peers to model resilience and provide real-time feedback. This context helps explain why the role of peer assistance in strengthening stress-coping capacities, as noted by Robinson et al. (2015), is particularly relevant in competitive swimming.

Our findings demonstrate a positive relationship between peer support and mental toughness in competitive swimming, aligning with previous research on social support in athletic settings. Such consistency with existing literature may be attributed to the fundamental human need for social connection and validation. This result echoes (Kumalasari’s, 2023) findings regarding academic resilience, possibly because both athletic and academic environments create performance pressures that activate similar social support mechanisms. It also reinforces the broader evidence on social support and mental toughness established by Jones et al. (2002). Similar to studies by Connaughton et al. (2010) and Dawson and Pooley (2013), which emphasized the importance of diverse social support sources, this study suggests that peer support serves as a key resource within swimming teams.

### 4.2 The relationship between self-efficacy, peer support, and mental toughness

Studies have shown that peer support can significantly predict self-efficacy, a result consistent with Zou et al. (2023) who demonstrated its positive effect on teenagers’ self-belief. This relationship is particularly evident in swimming teams, where close peer relationships markedly enhance self-efficacy. The underlying mechanisms may be understood through several factors specific to aquatic training settings, including the experience of wellbeing through shared training, secure attachment patterns within the team, and motivation through mutual encouragement, which collectively explain how peer support builds swimmers’ confidence. During swim training, peers frequently provide encouragement through verbal praise (Pan et al., 2021), while positive social interactions during practice enhance self-efficacy by boosting subjective wellbeing (Shaffer, 2005). Furthermore, consistent with Bowlby’s attachment theory, swimmers developing secure attachments to their training groups perceive stronger social support, creating psychological foundations that foster self-efficacy in aquatic environments (Wilson and Rodgers, 2004).

Research also confirms that self-efficacy significantly predicts mental toughness in swimmers, aligning with Brace et al. (2020) who observed similar effects in endurance athletes. In swimming, this relationship manifests through athletes’ enhanced capacity to cope with rigorous training and competitive pressures. Although Meggs et al. (2014) primarily attributed mental toughness to core self-evaluations, their inclusion of self-efficacy within this framework suggests its mediating role in resilience development, particularly relevant in swimming where sustained self-confidence determines athletes’ ability to persevere through demanding aquatic training.

### 4.3 The mediating role of self-efficacy

Building on existing evidence, our study explored a potential peer support pathway in swimming that is fully mediated. Peer support enhances the mental toughness of adolescent swimmers by strengthening their self-efficacy, these findings resonate with Shi et al. (2025), who demonstrated that social support influences youth athletes’ engagement through a chain mediation pathway. While their model extends to broader behavioral outcomes, both studies share a core mechanism: peer interactions foster efficacy beliefs, which in turn cultivate mental toughness.

While Fletcher and Sarkar’s (2014) dynamic model emphasizes resilience as a process shaped by protective factors, it lacks specificity on how peer interactions translate into cognitive adaptations. Within youth swimming settings, our findings extend this model by demonstrating that peer support operates through social cognitive mechanisms such as observational learning by emulating high-performing peers (Bandura, 2001), which collectively strengthen young swimmers’ self-efficacy beliefs. From the perspective of social cognition theory, this study investigates peer support as a catalyst in a cognitive behavioral process, where verbal encouragement from teammates enhances self-efficacy, thereby promoting goal directed efforts that ultimately strengthen mental toughness in young swimmers. Importantly, this integration provides empirical refinement to Freeman and Rees’ (2009) social support model, while their foundational work proposed a support to efficacy to outcomes pathway. The current study demonstrates, through the lens of social cognitive theory, how peer support enhances mental toughness in aquatic training environments. Specifically, environmental cues such as verbal encouragement during drills initiate cognitive appraisals centered on self-efficacy, which drive behavioral regulation manifested as goal-directed effort, ultimately fostering adaptive outcomes characterized by mental toughness in competitive young swimmers.

### 4.4 Practical applications and implications

To apply the findings, coaches can design simple, swim-specific activities that focus on building self-efficacy through peer interactions, ultimately improving mental toughness. First, during the training process, swimmers are divided into groups. They are filmed using smartphones or underwater cameras, and they discuss ways to optimize their techniques, translating peer support into incremental mastery experiences that directly strengthen self-efficacy (Bandura, 1997). Second, hold weekly team relays to boost unity, where swimmers must collaborate closely and verbally encourage each other. Track teamwork metrics like encouragement frequency and relay times in a shared training diary, reinforcing collective efficacy beliefs through visible progress toward shared goals. Third, simulate high-resistance conditions during relay tasks, where teammates rotate roles in overcoming physical challenges. After training, discuss how peer encouragement helped them persist under fatigue, explicitly framing these interactions as evidence that we can overcome tough conditions together, a core mechanism linking peer support to mental toughness via self-efficacy. These evidence-based intervention strategies may offer practical pathways for translating the fully mediated relationship into structured approaches, potentially providing coaches with actionable methods to support the development of self-efficacy and mental toughness in youth swimmers within authentic training scenarios.

### 4.5 Limitations and future research directions

Although this research has made certain contributions in both theory and practice, there are still some shortcomings. First, the cross-sectional design inherently restricts causal inferences between variables, and the analytical focus on a single pathway may not fully capture the multifaceted dynamic interactions inherent in social cognitive processes. Second, the findings are primarily situated within a specific cultural context, focusing on Chinese adolescent swimmers, who typically train in collectivist environments. This cultural specificity is noteworthy, as values and expressions of social interaction likely differ from those in individualistic cultures. Consequently, the support among peers may vary when dealing with stressful situations. Third, the study focused solely on positive peer interactions, overlooking potential negative peer influences that coexist in team environments. As the duration of interactions with peers grows, the chance of encountering adverse peer engagements intensifies.

To address these limitations, First, longitudinal studies can clarify whether peer support predicts mental toughness over time and how they may influence each other. Second, future research should test the model’s generalizability across sports and cultures where peer dynamics may differ. Finally, investigating how supportive and detrimental peer behaviors jointly shape self-efficacy and resilience could refine intervention designs. Addressing these gaps will enhance theoretical precision and practical relevance across diverse athletic populations.

## 5 Conclusion

The results indicate that the presence of peer support is significantly positively correlated with mental toughness. Self-efficacy was found to fully mediate this relationship. Therefore, it is suggested that coaches incorporate structured peer support activities into training to enhance athletes’ self-efficacy, thus strengthening their mental toughness.

## Data Availability

The raw data supporting the conclusions of this article will be made available by the authors, without undue reservation.
