# Correction: The mechanism of peer support on the mental toughness of adolescent swimmers: the mediating role of self-efficacy

**DOI:** 10.3389/fpsyg.2025.1733300

**Published:** 2025-12-02

**Authors:** Bingzhou Chen, Haixia Li, Ruiyun Zhang

**Affiliations:** 1 Graduate School of Education, Shandong Sport University, Jinan, China; 2 School of Sport Management, Shandong Sport University, Jinan, China; 3 College of Sports and Arts, Shandong Sport University, Jinan, China; 4 College of Physical Education and Health, East China Normal University, Shanghai, China

**Keywords:** peer support, mental toughness, self-efficacy, social cognitive theory, adolescent swimmers

There was a mistake in Figure 1 as published. The Mediation Model representing H1 was inaccurately represented as H2. The corrected Figure 1 appears below.

**Figure 1:**
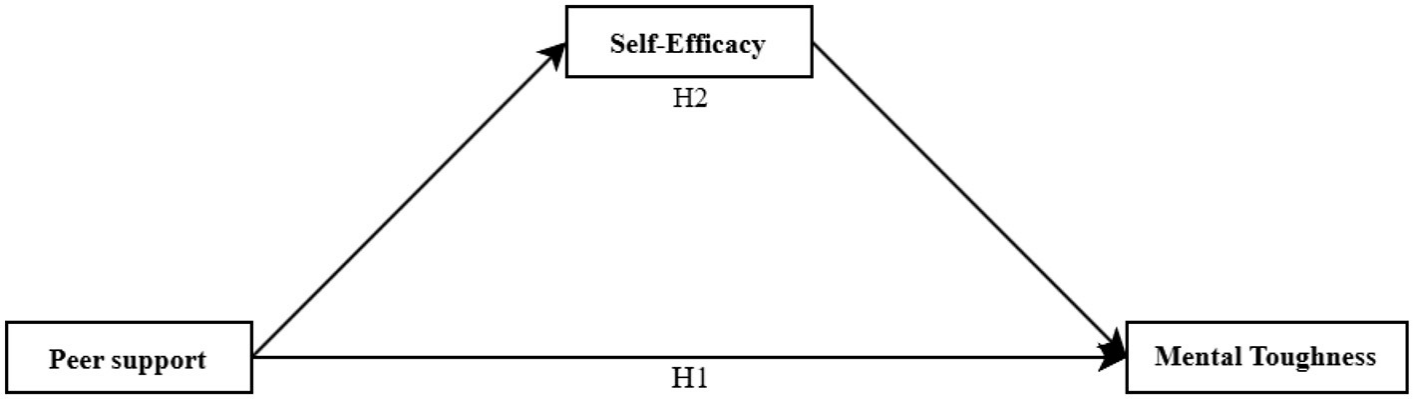
A hypothetical model of peer support affecting mental toughness.

The original version of this article has been updated.

